# Effectiveness of a Bivalent Recombinant Vaccine on the Production of Neutralizing Antibodies Against BoNT/C, BoNT/D, BoNT/CD e BoNT/DC in Bovines

**DOI:** 10.3390/vaccines13030299

**Published:** 2025-03-11

**Authors:** Ilenia Drigo, Luca Zandonà, Elena Tonon, Katia Capello, Luca Bano

**Affiliations:** 1 Istituto Zooprofilattico Sperimentale delle Venezie, SCT2 Sez. Territoriale di Treviso, 31020 Villorba, Italy; lzandona@izsvenezie.it (L.Z.); etonon@izsvenezie.it (E.T.); lbano@izsvenezie.it (L.B.); 2 Istituto Zooprofilattico Sperimentale delle Venezie, SCS4 Epidemiologia Analitica e Biostatistica, 35020 Legnaro, Italy; kcapello@izsvenezie.it

**Keywords:** bovine botulism, botulinum neurotoxins, recombinant vaccine

## Abstract

**Background/Objectives.** Bovine botulism, although relatively rare, presents significant economic losses due to high mortality rates and restrictions on livestock product trade. Vaccination remains the most effective strategy for preventing botulism-related mortality. This study evaluated the efficacy of a bivalent recombinant vaccine targeting the C-terminal portion of the heavy chain (Hc) of botulinum neurotoxin serotype C (BoNT/C) (Hc BoNT/C) and botulinum neurotoxin serotype D (BoNT/D) (Hc BoNT/D) in inducing neutralizing antibodies against these toxins and their mosaic variants BoNT/CD and BoNT/DC in cattle. This comparison aims to improve the design of an optimal recombinant vaccine for preventing bovine botulism caused by the most common serotypes. **Methods.** Twenty, four-month-old Holstein Friesian calves were randomly assigned to two groups of ten animals: vaccinated group and control group. Sera were collected at various time points to assess antibody titers using ELISA and neutralizing antibody titers using a mouse protection assay. Neutralizing antibody titers were compared to those obtained with a commercially available toxoid vaccine. **Results.** The recombinant vaccine elicited significant increases in anti-HcBoNT/C and anti-HcBoNT/D IgG antibody levels in vaccinated animals compared to controls animals with no adverse effects. Specifically, post-vaccination, the calves showed no local reactions (swelling, warmth) or behavioral changes suggestive of systemic illness. Neutralizing antibody titers against BoNT/C and BoNT/D were significantly higher in the recombinant vaccine group compared to the toxoid vaccine group. However, the recombinant vaccine showed lower neutralizing activity against BoNT/DC compared to the toxoid vaccine. **Conclusions.** The bivalent recombinant vaccine demonstrated promising immunogenicity in cattle, inducing high neutralizing antibody titers against BoNT/C and BoNT/D. While effective against these toxins, the lower efficacy against BoNT/DC highlights the need for further research to optimize the vaccine formulation, potentially by incorporating a BoNT/DC Hc component, to provide broader protection against bovine botulism.

## 1. Introduction

Although bovine botulism might be considered a rare disease, it nevertheless causes substantial economic losses for farmers. The primary economic impact stems from high mortality rates, compounded by restrictions on the commercialization of meat, milk, and dairy products, even from healthy animals within the same outbreak [1,2]. Botulism can arise from intoxication through the ingestion of preformed botulinum neurotoxins (BoNTs) in contaminated food or beverages, or from a toxico-infection resulting from BoNTs produced in the gut by previously ingested spores. One of the most common routes of feed contamination involves the accidental inclusion of an animal carcass in the forage [3,4,5,6,7]. There are seven distinct serotypes of BoNTs from A (BoNT/A) through G (BoNT/G) based on their antigenic properties [8,9]. In addition to these seven serotypes, a chimeric BoNT type H, also called FA or HA, and a putative novel type, called X, have been recovered in *Clostridium botulinum* (*C. botulinum*) strains previously classified as type B strains [10,11,12,13]. BoNT/A, B, E and F mainly cause human disease and are divided into subtypes based on their nucleic acid and amino acid sequence heterogeneity [14]. BoNT/C and D and their chimeric forms BoNT/CD and DC are the most common cause of botulism in animals [11,15,16,17]. In cattle, most of the reported outbreaks were caused by BoNT/C, DC, and D produced by *C. botulinum* group III whereas rare cases of type B and A botulism have been reported in The Netherlands, Brazil, the USA, and Israel [4,18,19,20,21,22,23]. Bovines are highly susceptible to BoNTs produced by *C. botulinum* group III on a per-kilogram basis. Indeed, it has been estimated that 1 g of BoNT type C can potentially kill up to 400,000 adult cows [7]. In BoNT/DC the light chain (Lc) and the amino-terminal domain of the heavy chain (H_N_) are of type D whereas the carboxyl-terminal domain (Hc) of the heavy chain are of type C. BoNT/CD on the contrary, consists of two-thirds of BoNT/C (Lc and H_N_) and one-third of Hc [24]. Recently, a new variant of BoNT/C has been described in Japan and has also been reported in France in human and pig outbreaks, respectively. The BoNT-gene of this new variant possesses the BoNT/DC gene in the Hc [17,25].

Conventionally, when botulism is suspected, the initial therapeutic intervention involves the intravenous administration of a polyvalent antitoxin. Since the antitoxin can only neutralize unbound botulinum toxin, it is crucial to administer it promptly at the onset of symptoms to be effective [26]. However, polyvalent antitoxins are currently not commercially available for animals in Europe. Several alternative therapeutic approaches have been evaluated, but none of them have proven effective in clinically recovering affected animals [26,27]. Vaccination has emerged as the most effective method for preventing botulism-related mortality, as supported by the extensive scientific literature [26,28,29,30]. Currently, no botulism vaccines are registered for use in Europe, and only limited stocks of toxoid-based traditional vaccines registered in non-EU countries are permitted in France. In Italy, a viable option involves the production of autogenous vaccines, utilizing toxoids derived from the supernatant of broth cultures of strains isolated during outbreaks [31,32,33]. However, toxoid-based vaccine production is time-consuming and carries inherent risks. Incomplete detoxification can pose a significant threat to animal health, while the handling of large quantities of active botulinum neurotoxins presents substantial safety concerns for manufacturers [33]. Recombinant DNA technology has emerged as a promising alternative to toxoid-based vaccine production and has been successfully employed for bovine immunization [29,30,34,35].

This study aimed to assess the humoral immune response of bovines to a bivalent BoNT/C and D recombinant vaccine in terms of neutralizing antibodies produced against BoNTs type C, D, and their mosaic variants C/D and D/C. The results were compared with those obtained with a traditional toxoid-based vaccine. The novelty of this study lies in comparing the neutralizing titer induced by a recombinant vaccine prepared with a portion of the Hc of non-mosaic BoNT/C and D also with respect to CD and DC mosaic forms. This comparison aims to optimize the development of an effective recombinant vaccine for the prevention of bovine botulism caused by the most prevalent serotypes. To the authors’ knowledge, no prior studies have evaluated vaccine efficacy also against these CD and DC mosaic forms.

## 2. Materials and Methods

### 2.1. Recombinant Vaccine

Recombinant peptides were produced at the Pasteur Institute under the supervision of Dr. Mazuet Christelle. HcBoNT/C, encompassing 425 amino acids from the C-terminal portion of the BoNT/C heavy chain, and HcBoNT/D, encompassing 413 amino acids from the C-terminal portion of the BoNT/D heavy chain, were used. They were produced as described in Stahl and coworkers using the *E. coli* BL21 (DE3) strain-carrying plasmid pET28a (Novagen, Gibbstown, NJ, USA) [36].

The final formulation of the recombinant vaccine contained 0.2 µg/kg HcBoNT/C and 0.2 µg/kg HcBoNT/D diluted in PBS and 4% aluminum hydroxide gel (Alu Gel-S^®^; SERVA Electrophoresis GmbH, Heidelberg, Germany) as adjuvant in a final volume of 2.5 mL. The vaccination protocol and dosage were determined based on results obtained with the same bivalent recombinant vaccine in Swiss Warmblood horses [36], which were vaccinated with 100 µg of peptides, given their average weight is 500 [37].

### 2.2. Experimental Groups

Twenty, four-month-old male Holstein Friesian veal calves, ranging from 110 to 130 kg of weight and housed in the same pen, were individually identified with ear tags and randomly assigned to two different experimental groups. The first group of 10 calves received three subcutaneous immunizations with the experimental recombinant vaccine at three-week intervals. The second group (control group) consisted of 10 calves: 5 subjects were inoculated at the same time as the first group with a solution containing PBS and 4% aluminum hydroxide gel (PAH subjects), and 5 served as untreated controls. Blood samples were collected via jugular venipuncture on the day of the first (T0, day 0), second (T1, day 21), and third (T2, day 42) vaccinations, as well as 21 days after the third vaccination (T3, day 63). Sera were stored at −80 °C until analysis. Within the first 48 h following each vaccination, the injection site was meticulously examined for swelling and warmth via visual inspection and palpation by the attending veterinarian. The farmer conducted daily evaluations of the animals’ general condition and behaviors, including appetite, willingness to move, and social interactions.

### 2.3. Enzyme Linked Immuno Assays

Ninety-six-well Maxisorp microtitration plates (Nunc GmbH & Co. KG, Langenselbold, Germany) were coated with 100 ng per well of HcBoNT/C or HcBoNT/D in 100 µL of carbonate buffer, 0.05 M, pH 9.6 (Sigma–Aldrich, Darmstadt, Germany), and allowed to incubate at 37 °C for 90 min. The plates were subsequently washed three times with 300 µL of phosphate-buffered saline containing 0.05% Tween-20 (PBST) (Sigma–Aldrich, Darmstadt, Germany) and nonspecific binding sites were blocked with 200 µL PBST added with 6% bovine serum albumin (BSA) (Sigma–Aldrich, Darmstadt, Germany) and 0.5 mM EDTA (Sigma–Aldrich, Darmstadt, Germany) for 1 h at 37 °C. After washing with PBST, 50 µL of sera diluted 1:100 in dilution buffer (DB, PBST plus 1% BSA and 0.5 mM EDTA), were added in each well in duplicate and plates were incubated 1 h at 37 °C. As a secondary antibody, a horseradish peroxidase-conjugated rabbit anti-bovine IgG (1:400) (Sigma–Aldrich, Darmstadt, Germany) was used in a quantity of 100 µL per well and incubated for 1 h 37 °C; the bound was detected by incubating the plates with 100 µL/well of ABTS peroxidase substrate (KPL, Gaithersburg, MD, USA) for 30 min in darkness. Absorbance values were read at 405 nm and the results were expressed in ELISA Units (EU)/mL (mean’s OD of the tested samples—OD of the negative control)/(OD of positive control—OD of negative control). The results of sera collected from bovines immunized with the recombinant vaccine were compared with those obtained from Holstein Friesian lactating cows with different parities vaccinated in 2013 with a commercial toxoid-based vaccine registered for use in cattle outside Europe. The vaccine was administered twice, four weeks apart, according to the leaflet instructions. Sera from these bovines were collected 4 weeks after the second inoculation, submitted to the Istituto Zooprofilattico Sperimentale delle Venezie (IZSVE) for diagnostic purposes and subsequently stored at −80 °C in the serum bank of the Diagnostic Veterinary Laboratory of Treviso. The vaccine leaflet does not specify if the toxoids contained in the vaccine derived from mosaic or non-mosaic BoNT/C and D. An indirect ELISA for the detection of antibodies in these sera was performed using BoNT/C or DC holotoxins as capture antigens produced in a dialysis apparatus [3].

### 2.4. Titration of Neutralizing Antibodies

Neutralizing antibody titers against BoNT/C, BoNT/CD, BoNT/D, and BoNT/DC serotypes in sera were determined using a mouse protection assay described by Hatheway et al. [38]. This assay evaluates the ability of sera to neutralize neurotoxins in vivo using CD-1 (Swiss) male mice weighing 18–22 g [38].

Botulinum neurotoxins used for titration were produced in dialysis apparatus [3] from a pure culture of different *C. botulinum* group III field strain subtypes (C, C/D, D, D/C) isolated from animal botulism outbreaks. Strain serotypes of those strains were assessed by a multiplex real-time PCR protocol [39,40] and the toxicity of each toxin batch, expressed as mouse lethal dose 50 (mLD50) per mL, was determined by titration in mice using the method of Reed and Muench [41].

The amount of toxin used in titrations, also referred to as a “test dose”, was calculated as the amount of each mosaic and non-mosaic toxin neutralized (50% end-point) by the BoNT/C (NIBSC, code 01/508) and BoNT/D (NIBSC, code 61/001) antitoxins [38].

To perform the titration, the estimated test dose of each toxin was mixed with serial 2-fold dilutions of sera in a gelatin-phosphate buffer from 1:2 to 1:256 to determine the highest dilution that neutralized the toxin (50% end-point). Mixtures were homogenized and incubated at room temperature for 1 h; subsequently, 500 µL of the mixture was injected by intra-peritoneal route at each mice of a group of 4. The mice were observed for 96 h and the end-point was determined by the death or survival of the animals. Mice survival indicated the presence of neutralizing antibodies in the tested serum and the comparison with the reference sera for botulinum type C and type D was used to quantify the antibodies in International Units (IU) per mL.

The results were compared with those obtained from testing sera from animals vaccinated with the traditional toxoid-based vaccine.

### 2.5. Statistical Analysis

The mean values of anti-BoNT/C and D IgG in calves vaccinated with recombinant bivalent vaccine were statistically compared to those of the control animals using linear mixed models. The groups (vaccinated vs. control) and the sampling time were the fixed effects of the models; additionally, the sampling time was included in the repeated effects part of the model, using a compound symmetry covariance structure. In order to allow the normality of the residuals, the log data transformation was applied to dependent variables (i.e., anti-BoNT/C and D IgG distributions). The Akaike Information Criterion (AIC) and the residual diagnostics were used to evaluate the goodness of fit of the models. A general linear model was adopted to compare antibody titers, at sampling time T2, among cattle vaccinated with recombinant bivalent vaccine (anti-BoNT/C and D IgG) and traditional toxoid-based vaccine.

Regarding the evaluation of neutralizing antibodies in calves vaccinated with bivalent recombinant vaccine, the comparison between the values pre-vaccination and after the second vaccination was considered; given that no variability was observed for the pre-vaccination values, one-sample Student’s t-test was adopted using the values of pre-vaccination as a constant.

Finally, the titers of neutralizing antibodies for cattle vaccinated with the recombinant vaccine compared to those immunized with the commercial toxoid vaccine were evaluated using the nonparametric k-sample test on the equality of medians, after having analyzed the distribution of values and only considering BoNT/C and BoNT/DC, given the limited availability of all samples.

All analyses were performed using SAS^®^ v.9.4

### 2.6. Ethics Statement

This study was carried out in accordance with national guidelines and EU Directive 2010/63/EU for animal experiments and was authorized by the Italian Ministry of Health (approval number No. 417/2017-PR of 18 May 2017). The animal study protocol was approved by the Ethics Committee of IZSVE (CE.IZSVE 12/2016).

## 3. Results

### 3.1. Evaluation of Anti-BoNT/C and Anti-BoNT/D IgG

**Figure 1:**
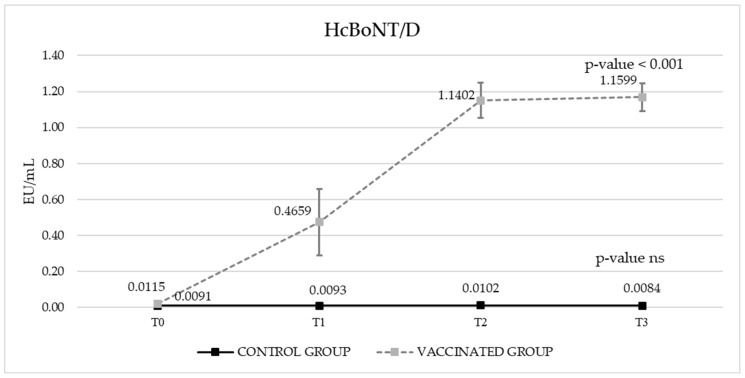
The total IgG titers against the HcBoNT/D of the vaccinated and control groups (EU/mL). For each group, the statistical significance of the overall trend has been reported. T0 = pre-vaccination; T1 = 21 days after the 1st vaccination; T2 = 21 days after the 2nd vaccination; T3 = 21 days after the 3rd vaccination. ns: not statistically significant.

**Figure 2:**
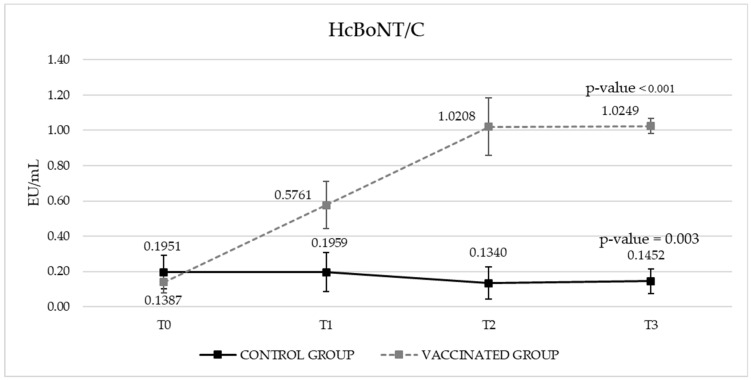
The total IgG titers against the HcBoNT/C of the vaccinated and control groups (EU/mL). For each group, the statistical significance of the overall trend has been reported. T0 = pre-vaccination; T1 = 21 days after the1st vaccination; T2 = 21 days after the 2nd vaccination; T3 = 21 days after the 3rd vaccination.

Post-vaccination, the calves exhibited no local reactions (swelling, warmth) or behavioral changes indicative of systemic illness. The results expressed in ELISA Units per milliliter (EU/mL) (Y-axis) are reported in Figure 1 and Figure 2 for antibodies against HcBoNT/D and HcBoNT/C, respectively. A marked increase on IgG titers against HcBoNT/C and HcBoNT/D was detected in all vaccinated bovines, starting from the first administration.

In particular, for HcBoNT/C, an overall significant difference (*p*-value < 0.001) was observed between control and vaccinated groups with lower mean EU values in the control one. Moreover, a significant interaction between groups and sampling time was highlighted (*p*-value < 0.001). The EU mean value of the control group showed a statistical difference between T2 and T0 (*p*-value = 0.006) and between T2 and T1 (*p*-value = 0.015); those differences could be due to some non-specific reaction observed in 3 to 10 subjects even before the first vaccination. As regards to the vaccinated bovines, the T2 sampling time showed significant differences both in T1 and T0 (*p*-value < 0.001), but no significant differences were found between T2 and T3 (Figure 2).

Regarding the IgG titer against HcBoNT/D, the overall difference between groups was confirmed for the vaccinated group (*p*-value < 0.001), followed by the significant interaction between group and sampling time (*p*-value < 0.001). In this case, the control group did not show any statistically significant difference among sampling times, whereas the vaccinated group confirmed statistical differences among both T1 and T2 and T2 and T0 (*p*-value < 0.001) and no differences between T2 and T3 (Figure 1).

No seroconversion was indeed observed when BoNT/C and BoNT/D holotoxins were used as capture antigens (IgG values remained below 0.3 EU/mL).

**Figure 3:**
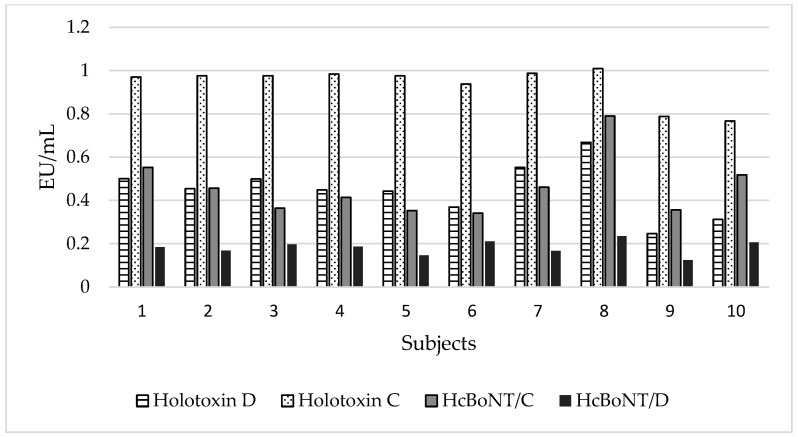
Antibody titers of calves vaccinated with the traditional toxoid-based bivalent vaccine. Different colors refer to the results obtained with a home-made indirect ELISA set up with the four capture antigens: holotoxin DC, holotoxin C, HcBoNT/C, and HcBoNT/D.

Antibody titers against holotoxins and recombinant peptides were evaluated also in 10 sera of bovines vaccinated twice with a traditional toxoid-based vaccine. The results are reported in Figure 3. All bovines showed a marked rise in antibodies titers against BoNT/C holotoxin, with a mean value of 0.937 EU/mL, whereas a weaker reaction was observed against BoNT/D holotoxin and the recombinant peptides HcBoNT/C. An almost absent reaction was observed against HcBoNT/D (mean EU/mL 0.18).

### 3.2. Evaluation of Neutralizing Antibodies in Calves Vaccinated with Bivalent Recombinant Vaccine

The capability of sera collected from animals vaccinated with the bivalent recombinant vaccine to neutralize BoNT/C, BoNT/D, BoNT/CD and BoNT/DC was evaluated by the mouse protection assay. The quantities of the NIBSC BoNT/C and BoNT/D reference antisera used to estimate the “test dose” of each toxin was calculated as described by Hatheway and coworkers [38] as follows: 0.053 IU/mL for BoNT/C, 0.027 IU/mL for BoNT/CD, 0.312 IU/mL BoNT/D, and 0.625 IU/mL for BoNT/DC, respectively.

Given the results obtained with the ELISA test and to limit the number of mice sacrificed, the test was initially performed only on sera collected before the vaccination (Pre-V) and after the second vaccination (Post-2nd) (Table 1, Supplementary Materials Tables S1–S4). No detectable neutralizing antibodies were evidenced in serum samples collected before the first vaccination. However, a significant rise in the neutralizing antibodies titer was evident after the second administration in all animals, most notably against BoNT/C and BoNT/D (*p* < 0.001) and, although lower, also against BoNT/CD (*p* = 0.017). On the contrary, no neutralizing antibodies were detected against BoNT/DC in nine to ten animals vaccinated with two doses. These antibodies appeared (1.372 ± 0.841 IU/mL), although low in concentration for most subjects, only after the third immunization (*p* = 0.02).

The titers of neutralizing antibodies against BoNT/C, BoNT/D, BoNT/CD, and BoNT/DC were also evaluated also in sera collected from animals immunized with two doses of the traditional toxoid vaccine. To optimize the use of limited serum, neutralization tests were primarily focused on serotypes C and DC, the most common serotype causing botulism in cattle. Any remaining serum was pooled to maximize the available volume for testing the tiers of the neutralizing antibodies against serotypes D and CD (Table 2).

**Figure 4:**
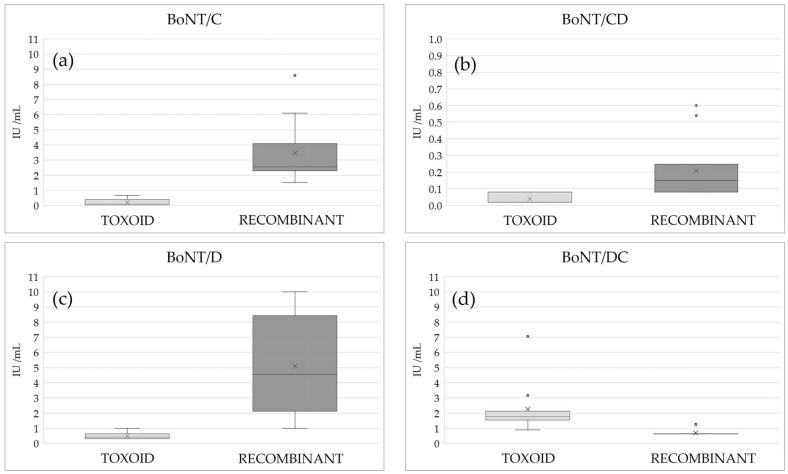
Neutralizing antibody titers against BoNT/C (**a**), BoNT/CD (**b**), BoNT/D (**c**), and BoNT/DC (**d**): comparison between traditional toxoid vaccine and recombinant vaccine 21 days after the second vaccination. (X = mean value; • = outlier value).

Neutralizing antibody titers against BoNT/C, BoNT/D, and BoNT/CD after the 2nd vaccination were significantly higher in cattle vaccinated with the recombinant vaccine compared to those immunized with the commercial toxoid vaccine (Figure 4). This difference was particularly pronounced for BoNT/C (*p*-value < 0.001, Figure 4a). Conversely, when bovine sera were tested against serotype DC, the toxoid vaccine demonstrated a higher capability in stimulating the production of neutralizing antibodies. This result was also confirmed when considering sera from bovine after three doses of the recombinant bivalent vaccine (mean value of 2.25 ± 1.79 IU/mL for toxoid vaccine vs. 1.37 ± 0.79 IU/mL for recombinant one after three doses; *p*-value = 0.002) (Figure 4d).

## 4. Discussion

Engineering of the BoNTs has played a crucial role in the production of safe vaccine candidates. The primary advantage of using recombinant techniques to produce protein- based vaccine in expression platforms is the possibility to yield highly purified antigens on a large-scale and at low costs [42]. Furthermore, the possibility to generate specific genetic sequences enables the development of vaccine antigens that retain the immunogenicity of whole BoNTs while excluding their toxicity [34,43]. However, there are currently seven BoNT toxinotypes and some of them contain subtypes that exhibit considerable amino acid sequence divergence. This high level of variability could potentially impact the ability of recombinant antigens to stimulate the production of efficient neutralizing antibodies [28,39,40]. The Hc of botulinum neurotoxin mediates binding to specific receptors on nerve cells surface, a critical step in toxin internalization [14]. This critical role makes this domain a significant target for the immune system and a key component in the development of safe and effective vaccines due to the presence of functional epitopes that elicit the production of neutralizing antibodies [14,44,45]. Previous studies investigating the BoNT Hc have also demonstrated its capacity to elicit high levels of protection [29,35,36,46,47,48].

In this study, we evaluate the efficacy of a recombinant vaccine targeting the C-terminal portion of the Hc of BoNT/C and BoNT/D in inducing a neutralizing immune response against homologous and mosaic CD and DC serotypes in cattle.

In our trial, vaccination significantly increased antibody titers against both HcBoNT/D and HcBoNT/C peptides (Figure 1 and Figure 2), with a notable absence of adverse clinical effects. Post-vaccination monitoring of calves revealed no local reactions (including swelling and warmth) or behavioral changes indicative of systemic illness, further demonstrating the vaccine’s safety.

Results showed that animals vaccinated with the bivalent recombinant vaccine developed high levels of neutralizing antibodies against BoNT/C, BoNT/D, with a milder response against BoNT/CD. A slight production of antibodies against BoNT/DC was only detected after third immunization (Figure 4). This result was expected as the amino acid sequence homology between the Hc of types D and DC is very low (37%), while the Hc terminal of type C share 77% homology with the Hc of type DC. Conversely, the slight neutralizing antibodies detected against BoNT/CD likely stem from the 93% homology of the amino acid sequences of Hc of this mosaic type D [49].

A comparative analysis revealed moreover that the recombinant vaccine generated substantially higher neutralizing antibody titers against BoNT/C and BoNT/D than the commercial toxoid (Figure 4a,c). Conversely, for the recombinant vaccine we observed a less robust response against BoNT/CD (Figure 4b) and minimal to no protective effect against BoNT/DC (Figure 4d), with the toxoid vaccine providing a higher protection against BoNT/DC. This difference in protection may arise from the potential presence of a DC mosaic toxin in the toxoid vaccine, a detail not disclosed in the product information leaflet.

These findings are consistent with prior research conducted in both bovine and other animal species. Stahl et al. (2009) [36] proved that the combination of the same HcBoNT/C and HcBoNT/D recombinant peptides used in this study elicited a protective immune response in horses, particularly against BoNT/D. Moreover, they found that horses vaccinated with the bivalent recombinant vaccine induced neutralizing antibody titers against C and D similar or superior to the ones obtained with the toxoid vaccine.

Cunha and coworker (2014) [30] tested, for the first time, a recombinant chimeric vaccine, designed to simultaneously target the Hc portions of BoNT/C and BoNT/D for prevention of botulism in cattle. The result demonstrated that the chimeric vaccine elicited neutralizing antibodies titer against BoNT/C equivalent to those produced by a conventional toxoid vaccine. However, this vaccine was able to induce a neutralizing antibodies titer against BoNT/D more than twice the levels achieved by the tested commercial traditional vaccine.

Moreira et al. (2020) [50] investigated the immunogenicity of a *E. coli* bacterins incorporating the HcBoNT/C and D peptides in cattle. They evaluated the recombinant vaccine’s ability to elicit anti- BoNT/C and D IgG and neutralizing antibodies in comparison with a commercial toxoid-based vaccine. The researchers concluded that the innovative vaccine produced superior immune response compared with the tested toxoid vaccine. Comparable results were obtained with the same vaccine formulation in buffaloes [35].

Notably, none of the aforementioned studies evaluated their recombinant vaccine’s ability to elicit protection against the mosaic serotypes CD and DC, which are the most frequent neurotoxins responsible for animal botulism [15]. Therefore, a gap currently exists in the literature regarding the evaluation of botulism vaccine protective capacity, whether toxoid or recombinant peptide-based, against these mosaic serotypes.

## 5. Conclusions

In summary, the bivalent recombinant vaccine tested in this study, containing fragments from the C-terminal Hc of BoNT/C and BoNT/D, effectively elicited high neutralizing antibody titers against the homologous neurotoxins, and, to a lesser extent, CD toxin in bovines after two administrations. While demonstrating superior efficacy against BoNT/C and D compared to a traditional toxoid vaccine, the recombinant vaccine showed lower protection against BoNT/DC intoxication. Considering that BoNT/C and BoNT/DC are the most common causative agents of bovine botulism [3,51,52,53,54], the development of a multivalent vaccine encompassing a BoNT/DC Hc is essential for achieving an effective and robust protection.

## Figures and Tables

**Table 1:** Neutralizing titers of sera collected before and after the immunization with the recombinant bivalent vaccine. Mean and standard deviation and *p*-value. * The minimum International Unit (IU) dose tested for each toxin was used as baseline for the statistical analysis. ND: not detected; NT: not tested.

BoNT	Pre-V		Post-2nd		Post-3rd		*p*-Value
	Mean * (IU/mL)	Std Dev	Mean (IU/mL)	Std Dev	Mean (IU/mL)	Std Dev	
BoNT/C	0.02	-	3.456	2.194	NT	/	<0.001
BoNT/D	0.3125	-	5.088	3.468	NT	/	<0.001
BoNT/CD	0.03	-	0.209	0.193	NT	/	0.017
BoNT/DC	0.625	-	ND	/	1.374	0.842	0.020

**Table 2:** Neutralizing titers of sera collected after two immunizations with the toxoid-based vaccine. Mean and standard deviation. * The minimum International Unit (IU) dose tested for each toxin was used as baseline for the statistical analysis.

BoNT	N° Sera Samples/Pool Tested	Neutralizing Titer (IU/mL)	
		Mean *	Std Dev
BoNT/C	10 sera	0.194	0.24
BoNT/DC	10 sera	2.26	1.80
BoNT/D	6 pools (21 sera in total)	0.49	0.26
BoNT/CD	7 sera	0.037	0.019

## Data Availability

The raw data supporting the conclusions of this article will be made available by the authors on request.

## Supplementary Materials

The following supporting information can be downloaded at https://www.mdpi.com/article/10.3390/vaccines13030299/s1. Raw data concerning titration of neutralizing antibodies are reported in Tables S1–S4.

## Abbreviations

The following abbreviations are used in this manuscript:

BoNT	Botulinum neurotoxin
BoNT/C	Botulinum neurotoxin serotype C
BoNT/CD	Botulinum neurotoxin serotype CD
BoNT/DC	Botulinum neurotoxin serotype DC
BoNT/D	Botulinum neurotoxin serotype D
BoNT/A	Botulinum neurotoxin serotype A
BoNT/B	Botulinum neurotoxin serotype B
BoNT/E	Botulinum neurotoxin serotype E
BoNT/F	Botulinum neurotoxin serotype F
BoNT/G	Botulinum neurotoxin serotype G
Hc	Heavy chain
Lc	Light chain
HcBoNT/C	Heavy chain of Botulinum neurotoxin serotype C
HcBoNT/D	Heavy chain of Botulinum neurotoxin serotype D
IU	International Units
EU	ELISA Units
