# Supplementary material for: Effectiveness of a Bivalent Recombinant Vaccine on the Production of Neutralizing Antibodies Against BoNT/C, BoNT/D, BoNT/CD e BoNT/DC in Bovines
Source: Vaccines (Basel). 2025 Mar 11;13(3):299. doi: 10.3390/vaccines13030299 (PMC11946303; doi:10.3390/vaccines13030299)
Supplement: Supplementary file 1 [file vaccines-13-00299-s001.zip › vaccines-3481242-supplementary.pdf]

			SERUM DILUTIONS (Mice survived)								
	**Bovine ID**	**GROUPS**	**ND**	**1:2**	**1:4**	**1:8**	**1:16**	**1:32**	**1:64**	**1:128**	**1:256**	**IU/ml**
**BoNT/C**	47	CONTROLS	0/4									<0,02
	48		0/4	-	-	-	-	-	-	-	-	<0,02
	49		0/4	-	-	-	-	-	-	-	-	<0,02
	50		0/4	-	-	-	-	-	-	-	-	<0,02
	58		0/4	-	-	-	-	-	-	-	-	<0,02
	52		0/4	-	-	-	-	-	-	-	-	<0,02
	54		0/4	-	-	-	-	-	-	-	-	<0,02
	55		0/4	-	-	-	-	-	-	-	-	<0,02
	57		0/4	-	-	-	-	-	-	-	-	<0,02
	59		0/4	-	-	-	-	-	-	-	-	<0,02
	51	RECOMBINAT VACCINE	4/4	4/4	4/4	4/4	4/4	4/4	1/4	1/4	0/4	3.42
	56		4/4	4/4	4/4	4/4	4/4	4/4	3/4	3/4	0/4	8.58
	60		4/4	4/4	4/4	4/4	4/4	4/4	4/4	1/4	0/4	6.1
	61		4/4	4/4	4/4	4/4	4/4	3/4	0/4	-	-	2.42
	62		4/4	4/4	4/4	4/4	4/4	2/4	0/4	-	-	1.92
	63		4/4	4/4	4/4	4/4	4/4	3/4	0/4	-	-	2.42
	64		4/4	4/4	4/4	4/4	4/4	4/4	1/4	0/4	-	3.06
	65		4/4	4/4	4/4	4/4	4/4	4/4	0/4	-	-	2.7
	66		4/4	4/4	4/4	4/4	4/4	1/4	0/4	-	-	1.52
	67		4/4	4/4	4/4	4/4	4/4	3/4	0/4	-	-	2.42
	9	TOXOID VACCINE	4/4	1/4	0/4	-	-	-	-	-	-	0.1
	12		0/4	-	-	-	-	-	-	-	-	<0,02
	2		4/4	4/4	3/4	1/4	0/4	-	-	-	-	0.34
	3		4/4	4/4	4/4	3/4	0/4	-	-	-	-	0.54
	4		0/4	-	-	-	-	-	-	-	-	<0,02
	8		4/4	1/4	1/4	0/4	-	-	-	-	-	0.1
	16		4/4	1/4	0/4	-	-	-	-	-	-	0.1
	14		4/4	4/4	4/4	4/4	0/4	-	-	-	-	0.68
	19		0/4	-	-	-	-	-	-	-	-	<0,02
	31		0/4	-	-	-	-	-	-	-	-	<0,02

Table S1. Titration of neutralizing antibodies against BoNT/C after second vaccination: result of the mouse bioassay and IU/ml calculation following the method described by Hatheway and Dang [38]. (-): not analyzed; IU: international unit; ND: not diluted.

			SERUM DILUTIONS (Mice survived)									
	**Bovine ID**	**GROUPS**	**ND**	**1:2**	**1:4**	**1:8**	**1:16**	**1:32**	**1:64**	**1:128**	**1:256**	**IU/ml**
**BoNT/CD**	47	CONTROLS	0/4	-	-	-	-	-	-	-	-	<0,03
	48		0/4	-	-	-	-	-	-	-	-	<0,03
	49		0/4	-	-	-	-	-	-	-	-	<0,03
	50		0/4	-	-	-	-	-	-	-	-	<0,03
	58		0/4	-	-	-	-	-	-	-	-	<0,03
	52		0/4	-	-	-	-	-	-	-	-	<0,03
	54		0/4	-	-	-	-	-	-	-	-	<0,03
	55		0/4	-	-	-	-	-	-	-	-	<0,03
	57		0/4	-	-	-	-	-	-	-	-	<0,03
	59		0/4	-	-	-	-	-	-	-	-	<0,03
	51	RECOMBINAT VACCINE	4/4	4/4	2/4	0/4	-	-	-	-	-	0.11
	56		4/4	4/4	0/4	-	-	-	-	-	-	0.08
	60		4/4	4/4	4/4	0/4	-	-	-	-	-	0.15
	61		4/4	4/4	4/4	0/4	-	-	-	-	-	0.15
	62		4/4	4/4	4/4	4/4	4/4	0/4	-	-	-	0.6
	63		4/4	4/4	4/4	4/4	3/4	0/4	-	-	-	0.54
	64		4/4	4/4	4/4	0/4	-	-	-	-	-	0.15
	65		4/4	4/4	4/4	2/4	0/4	-	-	-	-	0.15
	66		4/4	4/4	0/4	-	-	-	-	-	-	0.08
	67		4/4	4/4	0/4	-	-	-	-	-	-	0.08
	9	TOXOID VACCINE	0/4	-	-	-	-	-	-	-	-	<0,03
	12		0/4	-	-	-	-	-	-	-	-	<0,03
	2		4/4	4/4	0/4	-	-	-	-	-	-	0,08
	3		4/4	-	-	-	-	-	-	-	-	<0,03
	4		0/4	-	-	-	-	-	-	-	-	<0,03
	19		0/4	-	-	-	-	-	-	-	-	<0,03
	31		0/4	-	-	-	-	-	-	-	-	<0,03

Table S2. Titration of neutralizing antibodies against BoNT/CD after second vaccination: result of the mouse bioassay and IU/ml calculation following the method described by Hatheway and Dang [38]. (-): not analyzed; IU: international unit; ND: not diluted.

	Bovine ID	GROUPS	SERUM DILUTIONS (Mice survived)									
			ND	1:2	1:4	1:8	1:16	1:32	1:64	1:128	1:256	IU/ml
BoNT/D	47	CONTROLS	0/4	-	-	-	-	-	-	-	-	<0,3125
	48		0/4	-	-	-	-	-	-	-	-	<0,3125
	49		0/4	-	-	-	-	-	-	-	-	<0,3125
	50		0/4	-	-	-	-	-	-	-	-	<0,3125
	58		0/4	-	-	-	-	-	-	-	-	<0,3125
	52		0/4	-	-	-	-	-	-	-	-	<0,3125
	54		0/4	-	-	-	-	-	-	-	-	<0,3125
	55		0/4	-	-	-	-	-	-	-	-	<0,3125
	57		0/4	-	-	-	-	-	-	-	-	<0,3125
	59		0/4	-	-	-	-	-	-	-	-	<0,3125
	51	RECOMBINAT VACCINE	4/4	4/4	1/4	0/4	-	-	-	-	-	0.99
	56		4/4	4/4	4/4	4/4	1/4	0/4	-	-	-	3.98
	60		4/4	4/4	4/4	2/4	0/4	-	-	-	-	2.5
	61		4/4	4/4	4/4	2/4	0/4	-	-	-	-	2.5
	62		4/4	4/4	4/4	4/4	2/4	0/4	-	-	-	4.97
	63		4/4	4/4	4/4	4/4	4/4	2/4	0/4	-	-	10
	64		4/4	4/4	4/4	4/4	4/4	0/4	-	-	-	7.05
	65		4/4	4/4	4/4	4/4	4/4	1/4	0/4	-	-	7.9
	66		4/4	4/4	1/4	0/4	-	-	-	-	-	0.99
	67		4/4	4/4	4/4	4/4	4/4	2/4	0/4	-	-	10.00
	2-10	TOXOID VACCINE	4/4	4/4	1/4	0/4	-	-	-	-	-	0.99375
	6-21		0/4	-	-	-	-	-	-	-	-	< 0,3125
	20-32		0/4	-	-	-	-	-	-	-	-	< 0,3125
	1-12-13		0/4	-	-	-	-	-	-	-	-	< 0,3125
	3-4-5-7-8		4/4	1/4	0/4	-	-	-	-	-	-	0.496875
	9-14-15-16-17-18-19		4/4	1/4	0/4	-	-	-	-	-	-	0.496875

Table S3. Titration of neutralizing antibodies against BoNT/D after second vaccination: result of the mouse bioassay and IU/ml calculation following the method described by Hatheway and Dang [38]. (-): not analyzed; IU: international unit; ND: not diluted.

			SERUM DILUTIONS (Mice survived)									
	**Bovine ID**	**GROUPS**	**ND**	**1:2**	**1:4**	**1:8**	**1:16**	**1:32**	**1:64**	**1:128**	**1:256**	**IU/ml**
**BoNT/DC**	47	CONTROLS	0/4									<0,625
	48		0/4	-	-	-	-	-	-	-	-	<0,625
	49		0/4	-	-	-	-	-	-	-	-	<0,625
	50		0/4	-	-	-	-	-	-	-	-	<0,625
	58		0/4	-	-	-	-	-	-	-	-	<0,625
	52		0/4	-	-	-	-	-	-	-	-	<0,625
	54		0/4	-	-	-	-	-	-	-	-	<0,625
	55		0/4	-	-	-	-	-	-	-	-	<0,625
	57		0/4	-	-	-	-	-	-	-	-	<0,625
	59		0/4	-	-	-	-	-	-	-	-	<0,625
	51	RECOMBINAT VACCINE	0/4									<0,625
	56		0/4	-	-	-	-	-	-	-	-	<0,625
	60		0/4	-	-	-	-	-	-	-	-	<0,625
	61		0/4	-	-	-	-	-	-	-	-	<0,625
	62		0/4	-	-	-	-	-	-	-	-	<0,625
	63		0/4	-	-	-	-	-	-	-	-	<0,625
	64		0/4	-	-	-	-	-	-	-	-	<0,625
	65		0/4	-	-	-	-	-	-	-	-	<0,625
	66		0/4	-	-	-	-	-	-	-	-	<0,625
	67		0/4	-	-	-	-	-	-	-	-	<0,625
	10	TOXOID VACCINE	4/4	4/4	4/4	4/4	0/4	-	-	-	-	7.05
	12		4/4	0/4	-	-	-	-	-	-	-	0.88125
	18		4/4	0/4	-	-	-	-	-	-	-	0.88125
	1		4/4	4/4	0/4	-	-	-	-	-	-	1.7625
	17		4/4	4/4	0/4	-	-	-	-	-	-	1.7625
	32		4/4	4/4	0/4	-	-	-	-	-	-	1.7625
	13		4/4	4/4	0/4	-	-	-	-	-	-	1.7625
	20		4/4	4/4	0/4	-	-	-	-	-	-	1.7625
	19		4/4	4/4	0/4	-	-	-	-	-	-	1.7625
	31		4/4	4/4	3/4	0/4	-	-	-	-	-	3.175

Table S4. Titration of neutralizing antibodies against BoNT/DC after second vaccination: result of the mouse bioassay and IU/ml calculation following the method described by Hatheway and Dang [38]. (-): not analyzed; IU: international unit; ND: not diluted.
